# People with dementia as exercise video game testers: Gathering end‐user perspectives

**DOI:** 10.1002/alz.094290

**Published:** 2025-01-09

**Authors:** Erica Dove, Rosalie H. Wang, Kara K. Patterson, Arlene J. Astell

**Affiliations:** ^1^ University of Toronto, Toronto, ON Canada

## Abstract

**Background:**

Commercially available exercise video games (‘exergames') can be used by people with dementia with the right (human) prompting and support. However, more information is needed about what makes these systems and games technologically accessible for this population, considering their cognitive difficulties. This study explores what works and doesn’t work for people with dementia when introducing new exergame systems and games to broaden opportunities for physical activity.

**Methods:**

Thirty‐two people living with dementia (mean Montreal Cognitive Assessment score: 12.75/30) were recruited from four community‐based adult day programs in Canada. Participants were recruited as ‘game testers' once weekly for six weeks at each day program. Participants with dementia tried different exergame systems (e.g., Xbox Kinect, Nintendo Switch) and games (e.g., darts, boxing, dancing, etc.). Concurrently, gameplay video recordings and feedback via the talk‐aloud protocol and audio‐recorded post‐game group debriefs were collected. Data were analyzed descriptively and are currently undergoing analysis using behavioural coding software to examine in‐game prompts and player movements.

**Results:**

In total, 292 gameplay turns occurred across the sessions. Some participants declined to play certain games after watching their peers experience accessibility challenges. Common reasons for ending a turn (beyond completing the game objective) included boredom, frustration, confusion, fatigue, and soreness. Systems with handheld controllers (e.g., Nintendo Wii) were less accessible for participants than gesture‐based controls due to the need to push buttons and attend to the controller while simultaneously performing physical motions. Additionally, games requiring multiple coordinated movements and those not accommodating additional age‐related impairments (e.g., range of motion impairments, mobility devices) were not widely accessible to this diverse group of participants.

**Conclusions:**

This study highlights the importance of involving people living with dementia in game development. The findings reveal which elements of current exergames make them accessible or inaccessible for people living with dementia. These findings can help design new exergames to increase access to physical activity for people with dementia. The findings will also interest dementia service providers who want to use exergames to increase physical activity for people with dementia.

